# Orlistat Induces Ferroptosis in Pancreatic Neuroendocrine Tumors by Inactivating the MAPK Pathway

**DOI:** 10.7150/jca.83118

**Published:** 2023-05-21

**Authors:** Mujie Ye, Feiyu Lu, Jinhao Chen, Ping Yu, Yanling Xu, Na He, Chunhua Hu, Yuan Zhong, Lijun Yan, Danyang Gu, Lin Xu, Jianan Bai, Ye Tian, Qiyun Tang

**Affiliations:** Department of Geriatric Gastroenterology, Neuroendocrine Tumor Center, Jiangsu Province Hospital, The First Affiliated Hospital of Nanjing Medical University, Institute of Neuroendocrine Tumor, Nanjing Medical University, Nanjing, China.

**Keywords:** orlistat, FASN, pancreatic neuroendocrine tumors, ferroptosis, MAPK pathway

## Abstract

**Background:** Orlistat is an antiobesity drug approved by the US Food and Drug Administration (FDA) with potential antitumor activity against a few malignant tumors, however, whether orlistat affects the progression of pancreatic neuroendocrine tumors (pNETs) remains unknown.

**Methods:** Protein and mRNA levels of FASN were measured using western blotting (WB) and qRT-PCR. The effects of FASN and orlistat on cell proliferation were examined using CCK-8, colony formation, and EdU assays. The effects of FASN and orlistat on cell migration and invasion were tested using a transwell assay. A lipid peroxidation assay was used to explore the effects of orlistat on ferroptosis. The function of orlistat *in vivo* was determined by xenograft in nude mice.

**Results:** Based on the results of WB and qRT-PCR, FASN was significantly up-regulated in pNET cell lines and public database indicated increased expression of FASN correlated with poor prognosis for patients with pNET. CCK-8, colony formation, and EdU assays showed that knockdown of FASN or treatment with orlistat suppressed the proliferation of pNET cells. The transwell assay indicated that the knockdown of FASN or treatment with orlistat inhibited the migration and invasion of pNET cells. WB and the peroxidation assay showed that orlistat induced ferroptosis in pNET cells. Moreover, orlistat was also found to inhibit the MAPK pathway in pNETs. Furthermore, orlistat showed excellent anti-tumor effects in xenografts in nude mice.

**Conclusion:** Altogether, our study demonstrates that orlistat inhibits the progression of pNETs by inducing ferroptosis mediated by inactivation of the MAPK signaling pathway. Therefore, orlistat is a promising candidate for the treatment of pNETs.

## Introduction

Pancreatic neuroendocrine tumors (pNETs) are one of the tumors developing from pancreatic neuroendocrine cells and display high heterogeneity. In the past few decades, the incidence rate of pNETs among septuagenarians has increased to 16 cases per 100,000 1, taking advantage of developments in disease understanding and diagnostic technology. Currently, the classification standards of pNETs are mainly limited to Ki-67 proliferation and cell differentiation 2, and classification directly leads to therapeutic decision-making. Therefore, discovery of novel effective biomarkers is of great importance.

Lipid metabolism has been found to play an irreplaceable role in tumorigenesis in multiple tumor types 3. Tumor cells can facilitate lipogenesis, fatty acid intake, and fatty acid oxidation, thereby promoting energy and lipid accumulation. In the complex lipid metabolism process 4, fatty acid up-regulation is one of the most classically described metabolic processes in tumors, as it promotes tumorigenesis at multiple levels, including providing phospholipids for cellular membranes to sustain tumor cell proliferation and allowing the synthesis of important lipid mediators in certain tumors 5. It has been well documented that the up-regulation of diverse lipogenic enzymes facilitates this phenomenon 6. Fatty acid synthase (FASN) is a lipogenic enzyme that is up-regulated in multiple types of cancers, affecting many biological processes, and has been associated with survival curves and tumor grades 7. Interestingly, a recent study has found that the expression of FASN was related to ferroptosis in lung cancer 8, which opens up new horizons in cancer research.

Orlistat, a FASN inhibitor, has been shown to impair tumor cell proliferation and metastasis in several cancers, including colon, prostate, and lung cancer 9-14. Multiple mechanisms and targets are involved in the anticancer function of orlistat 15, however, studies on whether and how orlistat and FASN impact pNET tumorigenesis are still unclear.

In the present study, FASN was found to facilitate the development of pNETs by inducing ferroptosis-like cell death, which could be reversed by orlistat. This mechanism possibly provides new insight for the treatment of pNETs.

## Methods

### Cell lines and human tissues

The Human Pancreatic Nestin-Expressing ductal cell line (HPNE) was obtained from ATCC (CBP60857). The pNET cell line QGP-1 was registered in the JCRB cell bank (JCRB0183). Primary human pNET cells (referred to here as IPC619) were isolated from tumor tissues from patients diagnosed with pNETs, according to a previously described method 16. Both HPNE and QGP-1 cells were maintained in RPMI-1640 (Gibco, Carlsbad, CA, USA) supplemented with 10% fetal bovine serum (Yeasen, Shanghai, China) and 1% penicillin-streptomycin solution (HyClone, Logan, UT, USA). IPC619 cells were maintained in McCoy's 5A (Gibco, Carlsbad, CA, USA) supplemented with 10% fetal bovine serum. All cells were cultured in a 37 °C humidified CO_2_ incubator.

### Construction of stably transfected cell lines

The PLKO1 vector was used to construct FASN knockdown plasmids by Genomeditech (Shanghai, China). Lentivirus was packaged using 293T cells with PEI MAX (Polysciences, USA). Stably transfected cells were acquired after viral infection and filtration by adding puromycin into the medium.

### Quantitative Real-Time PCR (qRT-PCR)

TRIzol reagent (Life Technologies, USA) was used to extract total RNA from cell lines. Genomic DNA was then erased using a 5 × gDNA digester (Yeasen) at 42 °C for 2 min. Next, 4 x Hifair ® III SuperMix plus (Yeasen) was added to reversely transcribe RNA into complementary DNA at 37 °C for 15 min and 85 °C for 5 s. qRT-PCR was carried out using SYBR Green PCR master mix (Yeasen). Glyceraldehyde 3-phosphate dehydrogenase (GAPDH) served as an internal control for FASN. The primers used in the present study were: FASN-forward, 5'-AAGGACCTGTCTAGGTTTGATGC-3', FASN-reverse, 5'-TGGCTTCATAGGTGACTTCCA-3'; GAPDH-forward, 5'-ATCACCATCTTCCAGGAGCGA-3', GAPDH-reverse, 5'-CCTTCTCCATGGTGGTGAAGAC-3'. Data were analyzed using the 2^-△△ct^ method and visualized using GraphPad Prism 9 software.

### Western blotting (WB)

RIPA buffer containing a protease inhibitor (Beyotime, Nantong, China) was used to lyse the tissues or cells. Equal amounts of protein were separated using 10% sodium dodecyl sulfate-polyacrylamide gel electrophoresis (NCM Biotech, Suzhou, China) and then transferred onto nitrocellulose filter membranes (Millipore, USA). The membranes were blocked with 5% skim milk for 2 h. After washing twice with Tris-Buffered Saline Tween-20 buffer (Sevicebio, Wuhan, China), the membranes were incubated with primary antibodies, including anti-Fatty Acid Synthase (1:1000; Cell Signaling Technology), anti-GAPDH (1:5000; Proteintech), anti-xCT (1:1000; Cell Signaling Technology), anti-GPX4 (1:1000; Cell Signaling Technology), anti-CD71 (1:1000; Cell Signaling Technology), anti-p-ERK (1:1000; Cell Signaling Technology), or anti-ERK (1:1000; Cell Signaling Technology) overnight, followed by anti-rabbit IgG (1:2000; Cell Signaling Technology) or anti-mouse IgG (1:5000; Abmart) for 2 h. Lastly, the images were developed with ChemiScope 6100 (Clinx, Shanghai, China) after adding chemiluminescent substrate (NCM biotech).

### Cell Counting Kit-8 (CCK-8) assay

The CCK-8 assay was used to detect cell viability. 5 × 10^3^ QGP-1 cells or 2 × 10^3^ IPC619 cells were seeded in each well of a 96-well plate. After cultured for 0, 24, 48, and 72 h, 10 μL CCK8 reagent (Vazyme Biotech, Nanjing, China) was added to each well and cultured in an incubator for 2 h at 37 °C at each time point. The optical density at 450 nm was detected using a microplate reader.

### Colony formation assay

1 × 10^4^ QGP-1 cells or 1 × 10^4^ IPC619 cells were seeded into 6-well plates and cultured for 7 days in a medium containing 10% fetal bovine serum. The cells were then fixed with 4% paraformaldehyde for 30 min and stained with 0.2% crystal violet for 30 min. The number of colonies was counted by Image J.

### 5-ethynyl-2'-deoxyuridine (EdU) incorporation assay

Cells already planted in the 96-well plates were treated with 50 μM EdU medium (RiboBio, Guangzhou, China) at 37 °C for 2 h and then fixed with 4% paraformaldehyde for 30 min. Next, 0.5% Triton-X was used to permeabilize the cells and a 1 × Apollo reaction cocktail (RiboBio) was used to treat the cells for 30 min. The DNA contents of the cells were stained by treating the cells with Hoechst33342 for 30 min at room temperature. Images were visualized and photographed under a fluorescence microscope.

### Migration and invasion assays

For the migration assay, 2×10^5^ QGP-1 cells or 2×10^4^ IPC619 cells were seeded into the upper chamber of a transwell chamber (Corning, USA). For the invasion assay, 4×10^5^ QGP-1 cells or 4×10^4^ IPC619 cells were seeded into the upper chamber with a porous membrane constructed using Matrigel solution (BD, USA). The cells in the upper chamber were cultured in serum-free medium, while the lower chamber was filled with a medium containing 30% fetal bovine serum. After incubation at 37 °C for 48 h, the migrated or invaded cells were fixed using 4% paraformaldehyde for 30 min and then stained with 0.2% crystal violet for 30 min. The final photographs were taken under a microscope.

### Xenograft in nude mice

Male nude mice, 4-5 weeks old, were purchased from the Animal Center of Nanjing Medical University (Nanjing, China) and randomly grouped and maintained under pathogen-free conditions. Xenograft models were constructed by subcutaneously injecting 5 × 10^6^ QGP-1 cells into the armpit of mice and 10 mg/kg/day of orlistat was administered to one group of mice through gavage. After 4 weeks, the mice were sacrificed, and tumors were surgically excised 4 weeks after the injection. The tumors were then photographed and weighed; size was measured as 0.5 × length × width^2^.

### Immunohistochemistry

Tissues were embedded in paraffin and sectioned into 5 μm slices. Next, xylene and graded ethanol solutions were used to deparaffinize and hydrate the slices. The slices were boiled in 100 mM citrate buffer to unmask the antigen for 10 min, and 3% hydrogen peroxide was used to block the endogenous peroxidase activity. After blocking in 5% normal goat serum for 1 h, the slices were incubated with primary antibodies overnight at 4 °C and incubated the next day with secondary antibodies for 1 h. Finally, the slices were incubated with avidin-biotin complex for 1 h and stained with diaminobenzidine for 5 min. Images were taken under a microscope.

### Lipid peroxidation assay

5 × 10^5^ QGP-1 cells were planted per well in a 48-well plate overnight and then treated with DMSO or orlistat for another 24 h. Next, 50 μmmol/L C11-BODIPY581/589(Thermo Fisher, USA) was added into each well. The plate was then cultured at 37 °C for 20 min. The plate was then washed thrice with PBS and images were taken under a fluorescence microscope.

### Statistical analysis

All data were analyzed using the paired Student's t-test with the GraphPad Software. P values < 0.05(*), < 0.01(**), and < 0.001(***) were considered significant and the results are shown as means ± standard error. All experiments were repeated at least three times.

## Results

### Increased expression of FASN correlates with poor prognosis of patients with pNET

**Figure 1:**
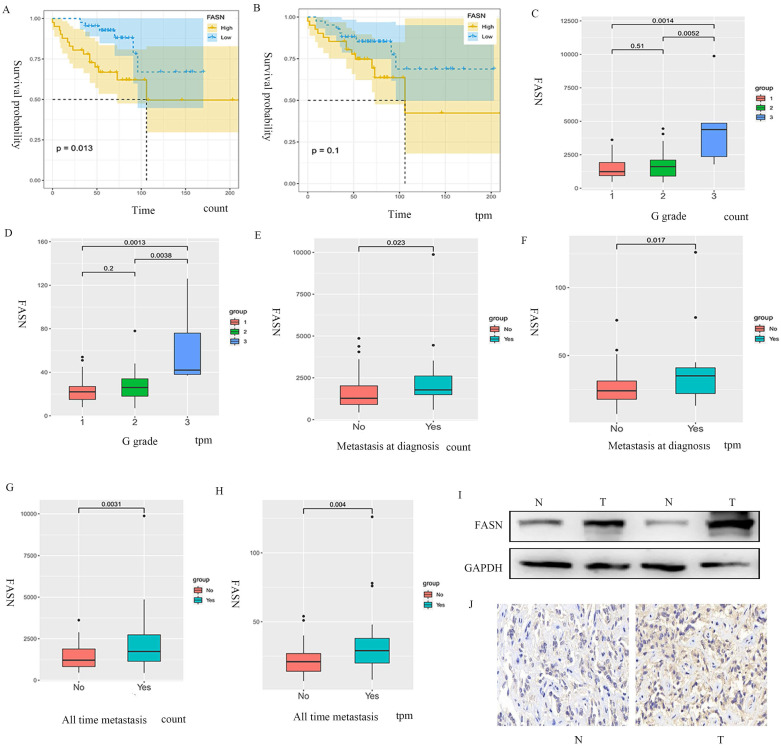
** FASN is correlated with poor prognosis in patients with pNETs.** (A, B) Kaplan-Meier analysis was performed according to the public database to analyze the effects of FASN on the overall survival of patients. (C-H) Further analysis was conducted to explore the relationship between FASN expression and G grade (C, D) and tumor metastasis (E-H). (I, J) Western blotting (WB) and immunohistochemistry were performed to assess FASN expression between pNET and normal tissue adjacent to pNET.

The samples extracted from the public database were divided into FASN-low-expression and FASN-high-expression groups based on gene expression, and Kaplan-Meier analysis was performed. The results indicated that the patients with pNETs with high FASN expression had lower overall survival than those with low FASN expression (Figures 1A, 1B). Furthermore, disease stage and tumor metastasis were also found to be related to FASN expression, as high FASN expression was consistent with worse disease stage and tumor metastasis (Figures 1C-1H). We also performed WB and immunohistochemistry to compare pNET tissue and normal tissue adjacent to pNET. The results showed that FASN expression was higher in the pNET tissue (Figures 1I, 1J). Thus, these results indicate that FASN is involved in the progression of pNETs and correlates with poor prognosis in patients with pNETs.

### Knockdown of FASN inhibits pNET cell proliferation

**Figure 2:**
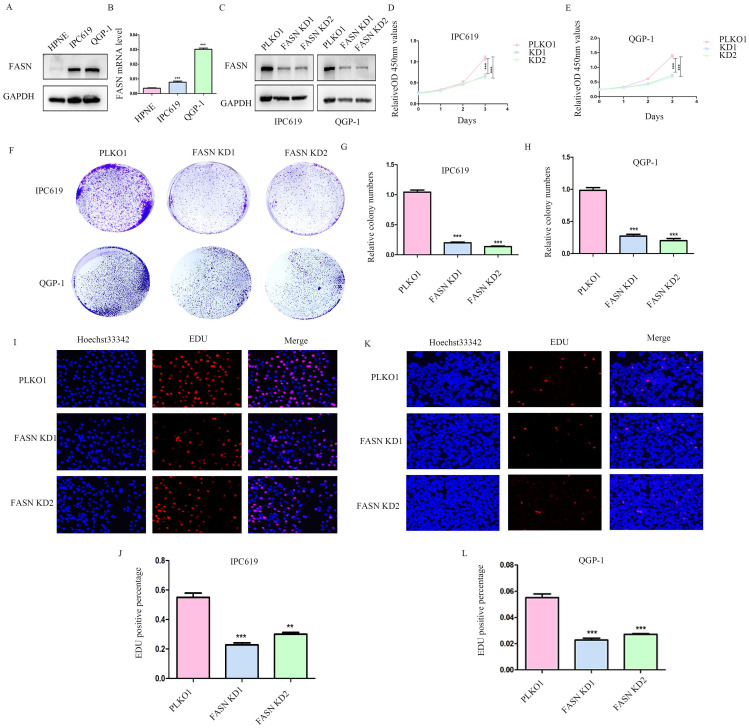
** Knockdown of FASN inhibits pNET cell proliferation.** (A, B) The FASN mRNA and protein expression levels in pNET cells were remarkably higher than those in normal human pancreatic cells. (C) Efficiency of construction for FASN knockdown cell lines. (D-L) CCK-8 (D, E), colony formation (F-H), and EdU incorporation (I-L) assays were performed to examine the effects of FASN on proliferation. **P<0.01, ***P<0.001.

To further determine the function of FASN in pNETs, we first examined the protein and mRNA levels in the normal human pancreatic cell line HPNE and in pNET cell lines. We observed that FASN expression was higher in pNET cells than in HPNE cells at both the protein and mRNA levels (Figures 2A, 2B). FASN was then knocked down in IPC619 and QGP-1 cells by transfection with a specific lentivirus. The protein level of FASN was assessed using WB (Figure 2C). Cell proliferation was obviously inhibited in stable FASN knockdown cells according to the results of CCK-8 (Figure 2D, 2E) and colony formation assays (Figures 2F-2H). An EdU assay was performed to measure the effect of FASN on cell proliferation. And the results verified that FASN knockdown suppressed the proliferation of pNET cells (Figures 2I-2L). Overall, these results demonstrate that FASN deficiency repressed the proliferation of pNET cells.

### Orlistat decreases pNET cell proliferation

**Figure 3:**
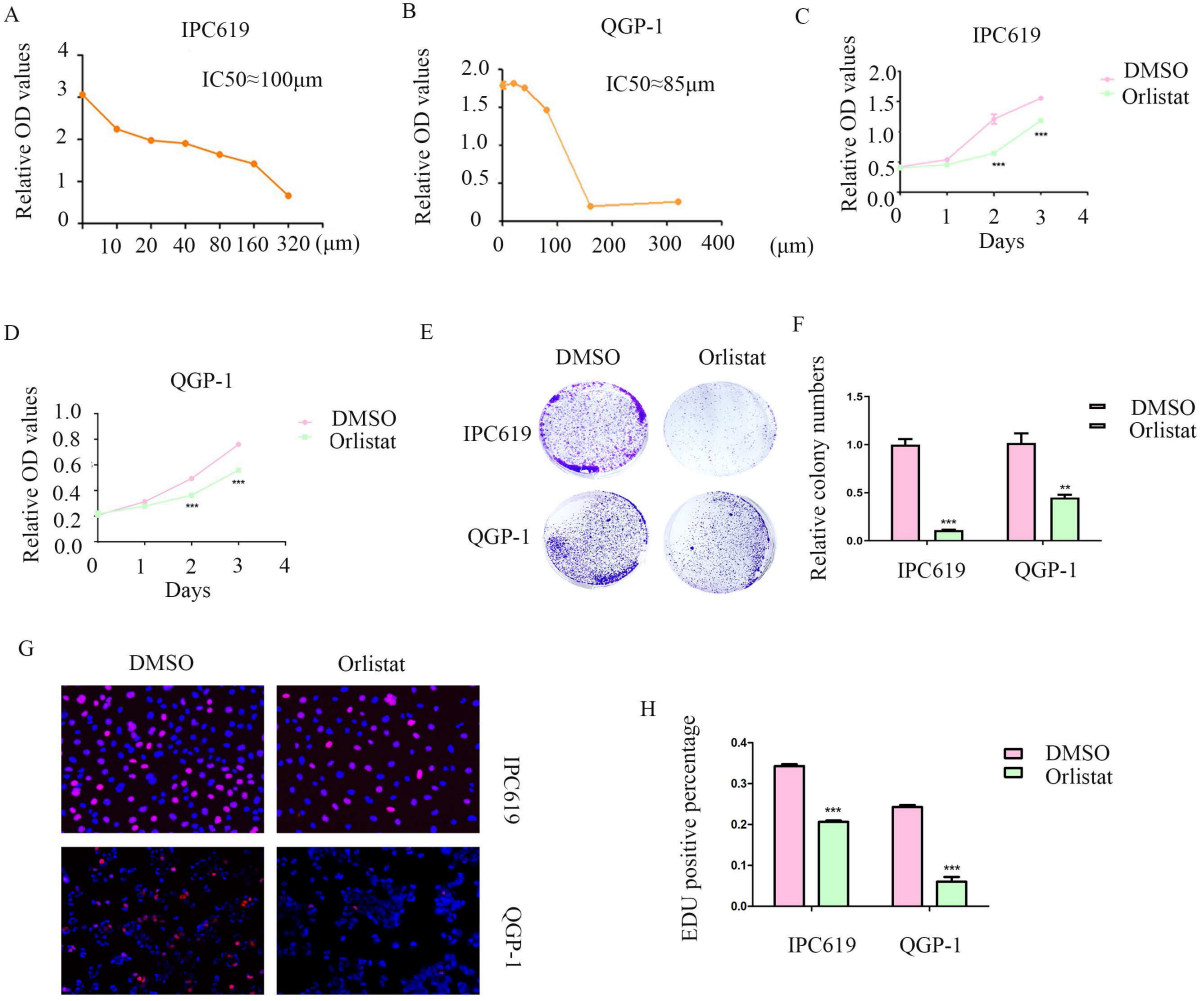
** Orlistat suppresses pNET cell proliferation.** (A, B) The half-maximal inhibitory concentration of orlistat was assessed in QGP-1 and IPC619 cells. (C-H) CCK-8 (C, D), colony formation (E, F), and EdU incorporation (G, H) assays were performed to examine the effects of orlistat on cell proliferation. **P<0.01, ***P<0.001.

Orlistat is a lipase inhibitor that mainly suppresses the activity of FASN. Therefore, we explored the potential anti-cancer function of orlistat in pNET cells by first testing the half-maximal inhibitory concentration on IPC619 and QGP-1 cell lines. And we selected 100 μM and 85 μM to treat IPC619 and QGP-1 cells, respectively, for 24 h before the experiments (Figure 3A, 3B). CCK-8 and colony formation assays showed that orlistat significantly inhibited the proliferation of pNET cells (Figures 3C-3F). An EdU assay was performed to explore the effects of orlistat on cell proliferation and also displayed orlistat decreased cell proliferation (Figures 3G, 3H). Taken together, these results indicate the anti-proliferative role of orlistat in pNET cells.

### FASN deficiency or treatment with orlistat inhibits cell migration and invasion in pNETs

**Figure 4:**
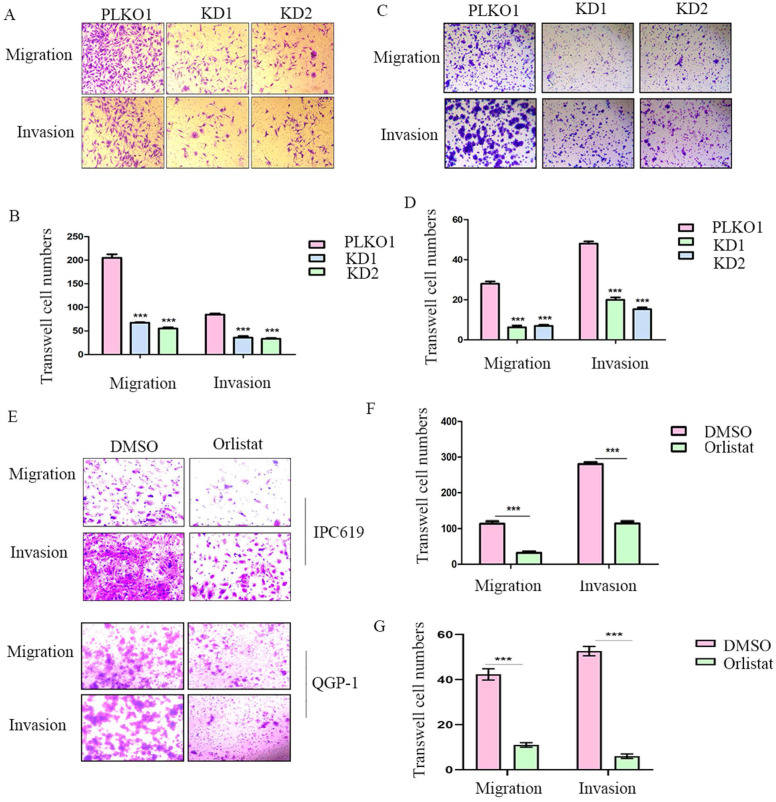
** Orlistat decreases pNET cell migration and invasion.** (A, B) Transwell assay was performed to assess the effects of FASN knockdown on the migration and invasion of IPC619 cells. (C, D) Transwell assay was conducted to assess the effects of FASN knockdown on the migration and invasion of QGP-1 cells. (E-G) The effects of orlistat on IPC619 and QGP-1 cell migration and invasion were assessed using transwell assay. ***P<0.001.

To further explore whether FASN affects cell migration or invasion of pNETs, we conducted transwell assays in IPC619 and QGP-1 cells with FASN silencing. The results showed that the knockdown of FASN in pNET cells inhibited cell migration and invasion (Figure 4A-4D). Transwell assays were then performed to test the effects of orlistat on migration and invasion, and it was confirmed that orlistat suppressed the migration and invasion of pNET cells (Figure 4E-4G). In conclusion, orlistat inhibits the migration and invasion of pNET cells by suppressing FASN.

### Orlistat suppresses pNET progression by inducing ferroptosis

**Figure 5:**
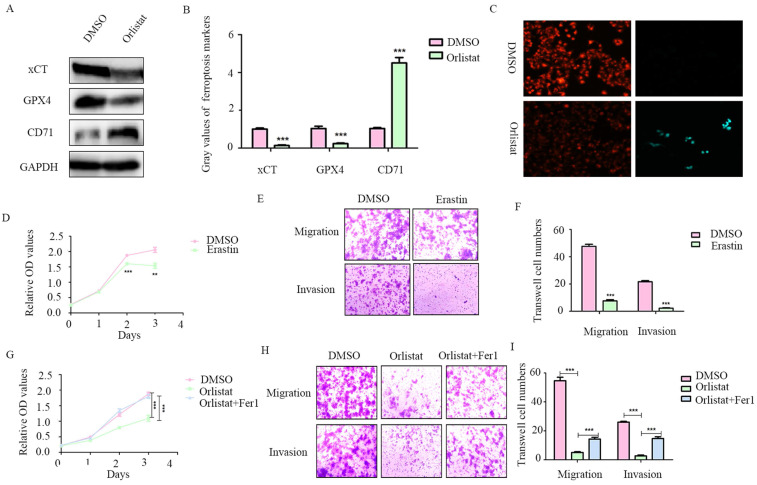
** Orlistat suppresses pNET progression by inducing ferroptosis.** (A, B) WB was performed to identify key molecules involved in ferroptosis, including xCT, GPX4, and CD71, in QGP-1 cells treated with IC50 of orlistat for 24 h. (C) Lipid peroxidation assay was performed to explore whether orlistat induced lipid peroxidation in QGP-1 cells. (D-F) CCK-8 and transwell assays were performed to detect whether erastin(10μm for 24h) affects the proliferation, migration, or invasion of QGP-1 cells. (G-I) CCK-8 and transwell assays were constructed to explore whether ferrostatin-1(Fer1, 10μm for 24h) could reverse the anticancer function of orlistat. **P<0.01; ***P<0.001.

A recent study reported that orlistat can induce ferroptosis-like cell death in lung cancer cells 14, we thus explored whether orlistat induces ferroptosis in pNET cells. WB was used to assess key molecules in the regulation of ferroptosis. A dramatic reduction in xCT and GPX4 and increase in CD71 levels were found in QGP-1 cells treated with orlistat compared to cells treated with DMSO (Figure 5A, 5B), indicating that orlistat induced ferroptosis. A lipid peroxidation assay showed that red fluorescence intensity was significantly reduced and green fluorescence intensity increased after QGP-1 cells were treated with orlistat, indicating that orlistat induced lipid peroxidation in pNET cells (Figure 5C). Moreover, we used erastin (an inducer of ferroptosis) to treat QGP-1 cells. Strikingly, the induction of ferroptosis suppressed the proliferation, migration, and invasion of QGP-1 cells (Figure 5D-5F). QGP-1 cells were also treated with ferrostatin-1, an inhibitor of ferroptosis, and the results showed that the tumor suppressive effect evoked by orlistat was inhibited to varying degrees after ferroptosis was impaired (Figure 5G-5I). Taken together, these results imply that, after orlistat treatment, ferroptosis is inevitable, resulting in the inhibition of the proliferation, migration, and invasion of pNET cells.

### Orlistat decelerates tumor progression through ferroptosis mediated by MAPK pathway

**Figure 6:**
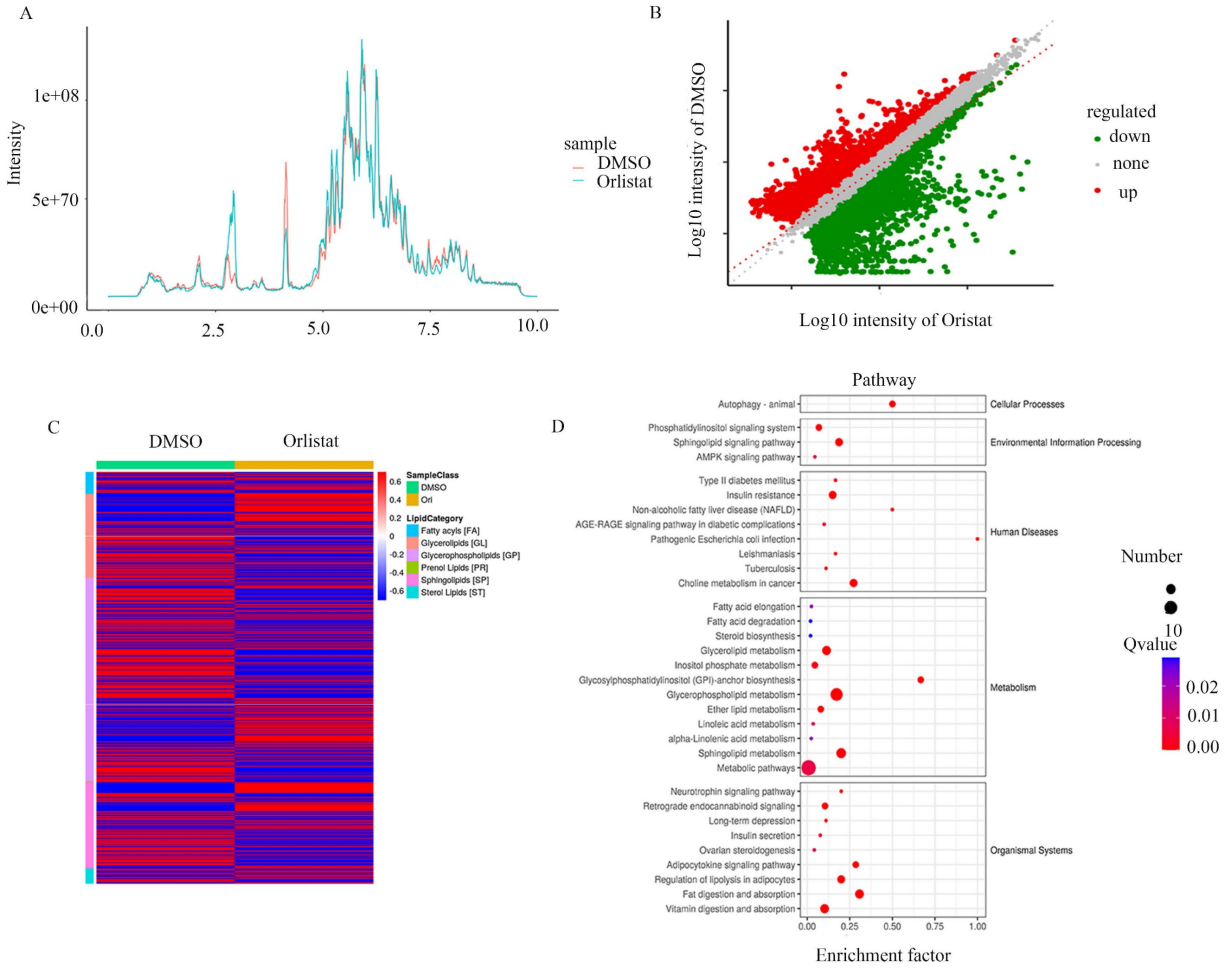
** Orlistat leads to changes of metabolic ions.** The metabolic ion densities of QGP-1 cells treated with DMSO and orlistat were measured. (B) The differential metabolic ions between QGP-1 cells treated with DMSO and orlistat were shown as volcanic plots. (C) Compared to DMSO, orlistat affected various processes related to lipid metabolism. (D) Orlistat affected a lot of pathways, particularly metabolic-related pathways.

To further explore the biological process impacted by orlistat, the metabolic ion density was measured (Figure 6A) and the differential metabolic ions are shown as volcanic plots (Figure 6B). The results suggest that orlistat affected various processes related to lipid metabolism (Figure 6C). Signaling pathways associated with ion enrichment were further detected, illustrating that orlistat mainly affected the metabolic-related pathways (Figure 6D).

**Figure 7:**
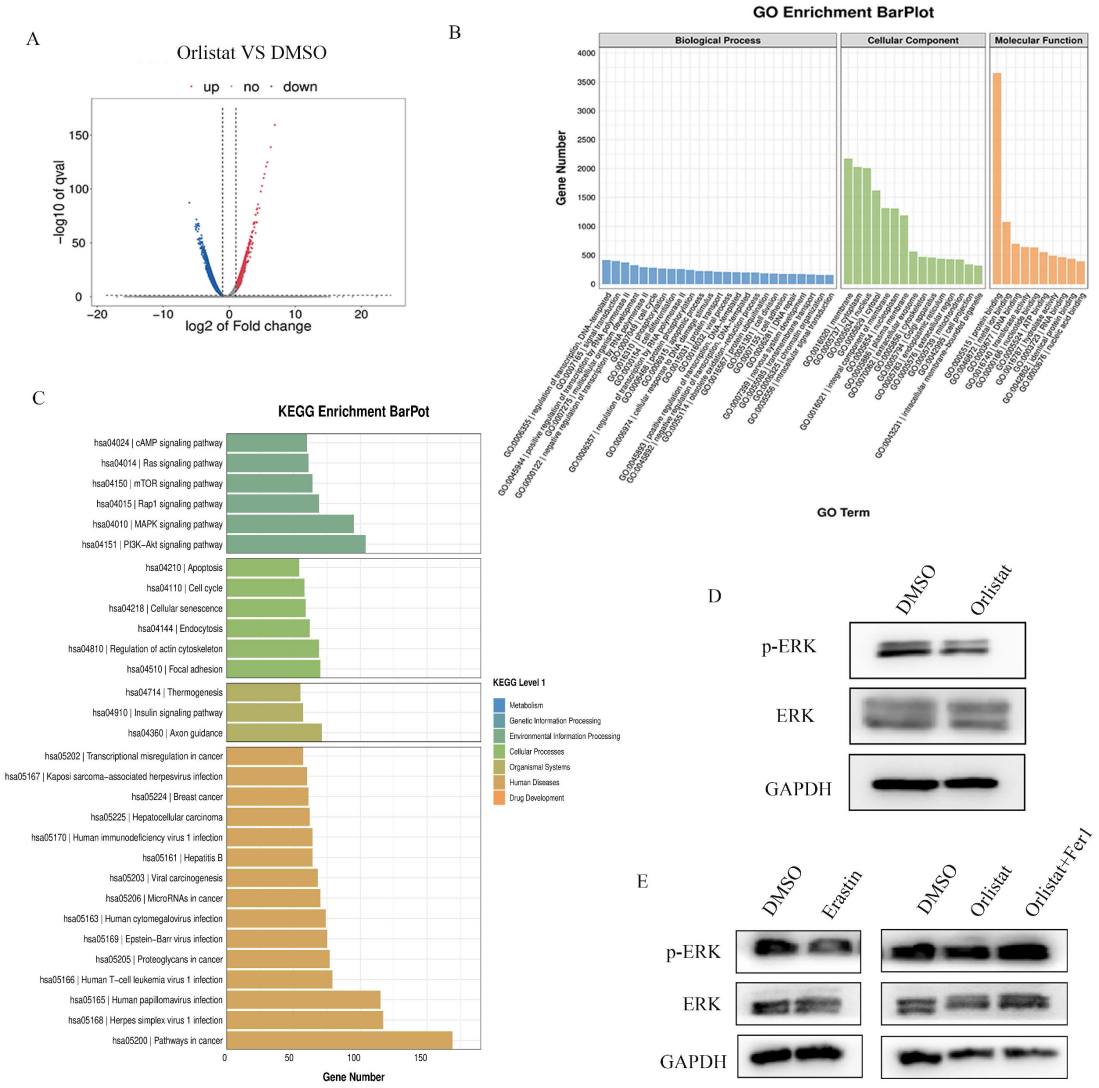
** Orlistat restrains tumor progression through ferroptosis mediated by the MAPK pathway.** (A) Differential genes between QGP-1 cells treated with DMSO and orlistat. (B) GO analysis showed that orlistat affects multiple biological processes. (C) KEGG enrichment results indicated that orlistat was involved in many pathways, including the MAPK pathway. (D, E) Key molecules of the MAPK pathway, including p-ERK and ERK, were examined using WB in QGP-1 cells treated with orlistat, erastin, and orlistat with ferrostatin-1.

RNA-seq assays were performed to further screen the underlying molecular mechanisms of how orlistat affects the progression of pNETs. Many differentially expressed genes were detected between QGP-1 cells treated with orlistat and DMSO (Figure 7A). GO enrichment analysis exhibited that orlistat may be involved in multiple metabolic processes (Figure 7B). Moreover, it was revealed that orlistat likely mediates its function via many signaling pathways, including the MAPK pathway (Figure 7C). Therefore, MAPK signal-related biomarkers, including MAPK and phospho-MAPK were examined using WB and it was shown that orlistat and erastin significantly reduced the expression of phospho-MAPK and that ferrostatin-1 reversed the reduction induced by orlistat (Figure 7D, 7E). Collectively, the above results clarify that orlistat may diminish the progression of pNETs through MAPK pathway-mediated ferroptosis.

### Orlistat abrogates pNET progression *in vivo* through ferroptosis accumulation

**Figure 8:**
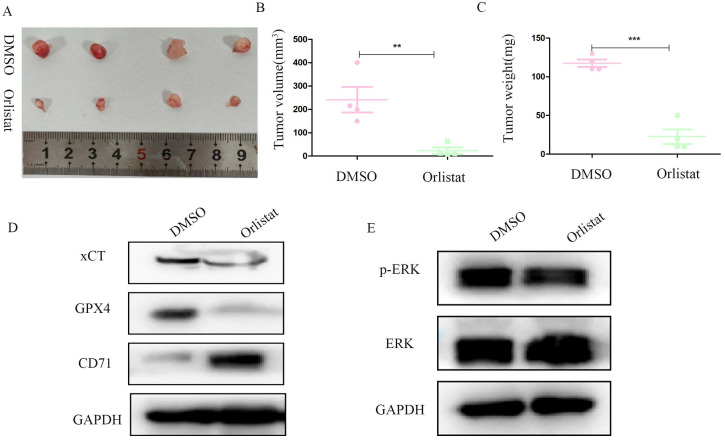
** Orlistat represses pNET progression *in vivo* by inducing ferroptosis.** (A) Nude mice subcutaneously injected with transfected QGP-1 cells were killed and tumors were extracted and photographed. (B, C) Tumors in the orlistat group had a lower volume and weight than those in the DMSO group. (D, E) Molecules related to ferroptosis and the MAPK pathway were examined in proteins extracted from tumors using WB. **P<0.01; ***P<0.001.

We constructed a nude mice xenograft model to further explore whether orlistat could inhibit the progression of pNETs *in vivo*. Similar with *in vitro*, *in vivo* results substantiated that the group treated with orlistat had lower volume and weight than the control group (Figure 8A-8C). WB was then performed and showed that the expression of xCT and GPX4 was significantly reduced in the group treated with orlistat, while that of CD71 increased, indicating that orlistat induced ferroptosis *in vivo* (Figure 8D). Moreover, the expression of phospho-MAPK was dramatically reduced in the group treated with orlistat (Figure 8E). In summary, these results highlight that orlistat attenuates the progression of pNETs *in vivo* by activating ferroptosis via inhibiting the MAPK pathway.

## Discussion

Orlistat is an anti-obesity drug registered in many countries that exerts its effect by suppressing the activities of gastrointestinal lipases, with FASN as the main lipase 17, 18. Although orlistat is a hydrophobic agent with low absorption capacity, it can prevent up to 30% absorption of lipids in dietary fats by disrupting lipid metabolism as the absorbed dose can sufficiently pass through cell membranes 19, 20. Moreover, in recent years, several interesting studies have reported the anti-tumor function of orlistat. For example, Ewa et al reported orlistat reduces proliferation and enhances apoptosis in human pancreatic cancer cells 21. Amira and colleagues investigated the possible anticancer activity of rutin and orlistat which are both safely used clinically in humans against two breast cancer models and the pancreatic cancer cell line 22. In this study, we found that administration of orlistat suppressed the progression of pNETs both *in vitro* and *in vivo*.

FASN, the main lipase suppressed by orlistat, has been reported in association with the cell cycle progression of malignant tumors and the activation of signaling pathways, including the PI3K-AKT and the MEK/ERK pathways 23. The up-regulation of FASN has been commonly considered a carcinogenic event. In our study, we first performed Kaplan-Meier analysis using sample data from a public dataset, and the results from the dataset showed that the patients with pNETs with high FASN expression had more severe overall survival. We further identified that FASN expression was correlated with the tumor grade and metastasis of pNETs, and FASN was significantly up-regulated in pNET cells. We then constructed stably FASN knockdown cells and verified that knockdown of FASN inhibited cell proliferation, migration, and invasion of pNETs, indicating that orlistat may have effects on pNETs. Further experiments showed that orlistat also attenuated the proliferation, migration, and invasion of pNET cells.

In recent years, ferroptosis has been found to be a type of controlled cell death in tumor formation in various cancers, including pancreatic, breast, and hepatocellular cancer 24-29. An increasing number of studies have attempted to use ferroptosis stimulation for cancer therapy 30, 31. GPX4 is responsible for the accumulation of lethal lipid peroxides given that GPX4 catalyzes lipid peroxides by activating the activity of phospholipid peroxidase 32, 33. Knockdown of GPX4 could result in ferroptosis and an increase in lipid reactive oxygen species, causing cell death in renal carcinoma cells 34. Moreover, erastin, a ferroptosis accelerator, can be combined with many drugs such as cisplatin, temozolomide, and adriamycin in cancer therapy 35. In this study, we sought to explore whether orlistat induced ferroptosis in pNETs, as it has been reported that orlistat can induce ferroptosis-like cell death in lung cancer cells. The results indicated that, after treatment with orlistat, xCT and GPX4 protein expression in QGP-1 cells was reduced, while CD71 expression was increased. Besides, the lipid peroxidation assay showed that orlistat induced lipid peroxidation in pNET cells. Taken together, these results demonstrated that orlistat induced ferroptosis in pNETs both *in vitro* and *in vivo*. To further detect the proportion of induced ferroptosis in orlistat-treated pNETs, we constructed a reverse experiment using the ferroptosis inhibitor ferrostatin-1. The anti-tumor effect of orlistat was thus dramatically reversed, which meant that the induction of ferroptosis accounted for much of orlistat's anti-tumor function. However, the specific mechanism by which orlistat induces ferroptosis in pNETs requires to be explored in further studies.

In addition, RNA-seq was performed to identify the mechanism underlying orlistat-induced suppression of pNET progression. Our results demonstrated a close association between orlistat and the MAPK pathway. Using WB, we found that the expression levels of phospho-MAPK were significantly reduced after treatment with orlistat *in vitro* and *in vivo*. Notably, after treatment with ferrostatin-1, the reduction in phospho-MAPK expression induced by orlistat was significantly reversed. These results indicate that orlistat inactivated the MAPK pathway in pNETs and that ferroptosis might be involved, which also deserves further exploration.

In conclusion, our study demonstrated that treatment with orlistat suppressed the expression of xCT and GPX4, increased the expression of CD71, thus facilitated lipid peroxidation and ferroptosis in pNET cells, inhibiting pNET progression mediated by the MAPK pathway. In summary, these findings may reflect novel orientations for the treatment of pNETs using orlistat and provide more possibilities for clinical application.
